# Direction of screw insertion for internal fixation plate in distal femoral osteotomy: Evaluation using axial computer tomography imaging

**DOI:** 10.1051/sicotj/2025066

**Published:** 2026-01-28

**Authors:** Fumiyoshi Kawashima, Ryuichi Nakamura, Akira Okano, Kaori Matsumoto, Jun Oike, Koji Kanzaki

**Affiliations:** 1 Department of Orthopaedic Surgery, Showa Medical University Fujigaoka Hospital 1-30 Fujigaoka, Aoba Ward, Yokohama Kanagawa 227-8501 Japan; 2 Joint Preservation and Sports Orthopaedics Center, Harue Hospital 65-7 Haruecho Haribara, Sakai Fukui 919-0476 Japan

**Keywords:** Bone screws, Tomography, X-Ray computed, Osteotomy, Distal femoral osteotomy, Plate fixation

## Abstract

*Purpose:* In distal femoral osteotomy (DFO), using longer distal screws in fixation plates may improve stability. This study examined the insertion direction of three distal screws at the horizontal cross-section to determine if posterior angulation enables deeper placement. *Methods:* Forty-seven varus knees that underwent DFO were included (medial closed-wedge DFO [MCWDFO], 30 knees; lateral closed-wedge DFO [LCWDFO], 17 knees). Postoperative plain CT images were obtained from a plane parallel to the three distal screws, with the most distal screw designated as A, the anterior of the second distal row as B, and the posterior of the second distal row as C. For each case, a curve passing through the center of the bony cortex on the cross-section parallel to each screw and over its entire length was drawn, and the curve and the lower edge of the screw were projected onto a graph. The maximum angle at which the lower edge of each screw touches the intercondylar region without interfering with the intercondylar region was designated as (AnA), (AnB), and (AnC) for A-, B-, and C-screws, respectively. The angle between the line connecting the insertion points of the B- and C-screws on the plate and the tangent line to the medial and lateral bony cortex was designated as (AnP). *Results:* In the MCWDFO group, the mean values for each parameter were AnA, 10.9 ± 5.4; AnB, 27.0 ± 4.2; AnC, 9.2 ± 3.4; and AnP, −2.6 ± 6.9. In the LCWDFO group, the mean values for each parameter were AnA, 18.2 ± 6.9; AnB, 30.4 ± 7.1; AnC, 16.1 ± 7.2; AnP, −0.2 ± 6.1°. *Conclusions*: The medial surface is inclined compared to the epicondylar axis and posterior condyle, usually resulting in plate positioning that is parallel to the placement surface. The optimal screw insertion from the anterior to posterior was generally achieved; however, there was still room for posterior angulation margins of 9–11° for A- and C-screws and approximately 27° for the B-screw. In contrast, the lateral surface is flatter with less inclination, causing anterior plate placement and wider posterior angulation – approximately 16–18° for A- and C-screws and 30° for the B-screw – allowing a greater range of posterior swing than the medial side.

## Introduction

Lateral closed wedge distal femoral valgus osteotomy (LCWDFO) [1, 2] and medial closed wedge distal femoral varus osteotomy (MCWDFO) [3–6] are surgical procedures for young, highly active patients with degenerative changes or cartilage damage in either the medial or lateral compartment and a deformity in the distal femur. Although internal fixation is required after osteotomy, the femur is susceptible to rotational forces due to the hip joint. Unlike the tibia, the femur lacks stabilizing mechanisms such as the patellar tendon and tibiofibular ligament, making it difficult to achieve mechanical stability after osteotomy [7]. Therefore, a stronger internal fixation is required.

Previously, there were no internal fixation devices that were specifically designed for DFO, and some reports have described the crude use of proximal tibia plates [7, 8]. However, locking plates specifically developed for distal femoral osteotomy (Tris Medial DFO and Lateral DFO Plates; Olympus Terumo Biomaterials, Tokyo, Japan) have been recently introduced in Japan and are now widely utilized. These plates are anatomically constructed and are designed to allow for long screw insertion distal to the osteotomy site [9]. While this is a well-designed implant, to our knowledge, we have not found any reports examining the direction of screw insertion in the horizontal cross-section of the distal osteotomy site.

Considering the fixation strength of devices, it is important that the distal screw can be inserted as long as possible for maximum purchase, and it is desirable to insert the screw from the insertion site toward the posterior end of the contralateral condyle. However, if inserted too far posteriorly, there is a risk of perforating the intercondylar space and breaching the articular surface. The purpose of this study was to measure the direction of insertion in the horizontal cross-section of the three distal screws of Tris DFO plates used in MCWDFO and LCWDFO, and to examine whether it is possible to position the path of the screws further posteriorly than currently recommended. The possibility of longer distal screw insertion may contribute to the development of new implant designs.

## Materials and methods

### Patient selection and exclusion criteria

**Figure 1:**
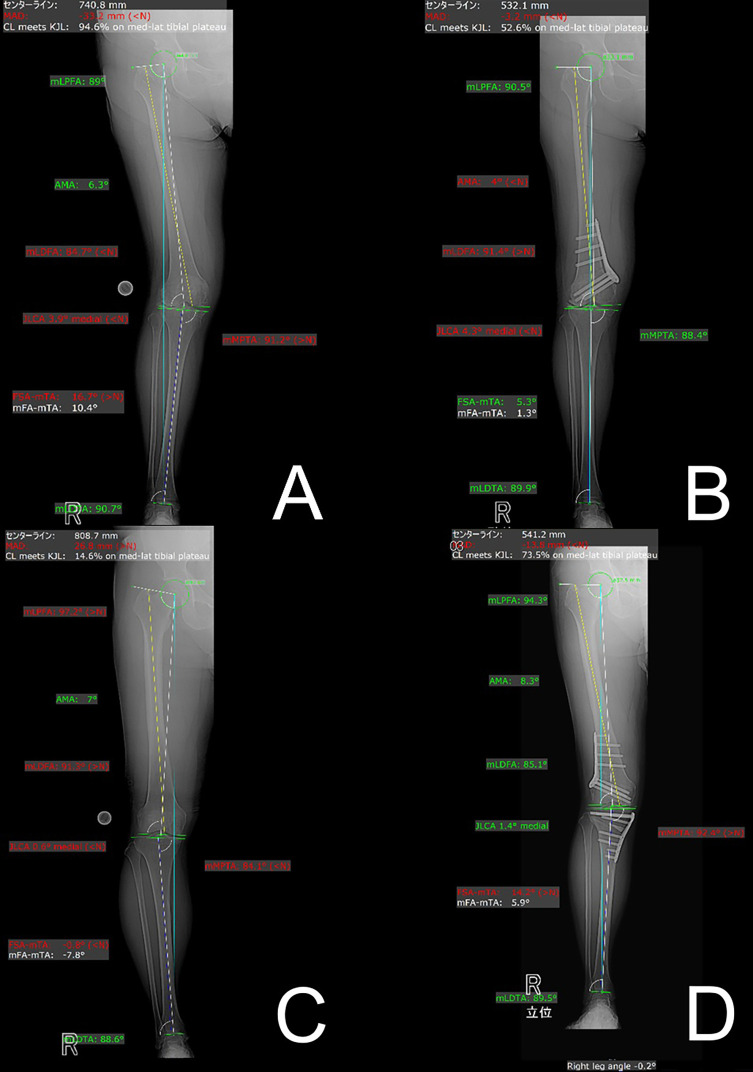
A. Preoperative full-length standing radiograph of the lower limb after MCWDFO for genu valgum. The mLDFA was 84.7°, demonstrating valgus alignment originating from the distal femur. B. Postoperative full-length standing radiograph of the lower limb after MCWDFO. The mLDFA was corrected to 91.4°, and the overall valgus alignment was corrected. C. Preoperative full-length standing radiograph of the lower limb after LCWDFO for genu varum. The mLDFA was 91.3° and mMPTA was 84.1°, demonstrating varus alignment originating from the distal femur and proximal tibia. D. Postoperative full-length standing radiograph of the entire lower limb after LCWDFO. The mLDFA was corrected to 85.1° and mMPTA was corrected to 92.4°, and the overall varus alignment was corrected.

This retrospective study was approved by the (blinded for review) Institutional Review Board (approval number: blinded for review) and (blinded for review) (approval number: blinded for review). The study included 26 patients (30 knees, 6 males, and 24 females) who underwent MCWDFO for distal femoral valgus knees (Figures 1A, 1B) and 17 patients (17 knees, 6 males, and 11 females) who underwent LCWDFO for varus knees (Figures 1C, 1D) at both hospitals between April 2022 and March 2025. Eight knees in the MCWDFO group and 16 knees in the MCWDFO group underwent double-level osteotomy combined with simultaneous high tibial osteotomy (HTO). Distal femoral valgus was defined as a hip-knee-ankle angle (HKA angle) of 3° or more on full-length standing plain radiographs in anteroposterior view [10], mechanical lateral femoral angle (mLDFA) of 85° or less, and a mechanical medial proximal tibial angle (mMPTA) of 85°–90°. Distal femoral varus was defined as an HKA of 5° or more and an mLDFA of 90° or more [11, 12]. Diagnoses excluded patients with previous knee ligament reconstruction, spinal fusion, and hip joint surgery.

### Radiographic evaluation

Computed tomography (CT) scans were obtained with a CT scanner (Fuji SCENARIA, Tokyo, Japan; 120 kVp: 500 mA, 512 × 512 matrix; slice thickness, 2.0 mm). mLDFA was measured on pre- and postoperative full-length standing plain radiographs. In addition, postoperative plain CT scans were performed to obtain the transverse sections from the most distal screw to the level of the third distal screw.

**Figure 2:**
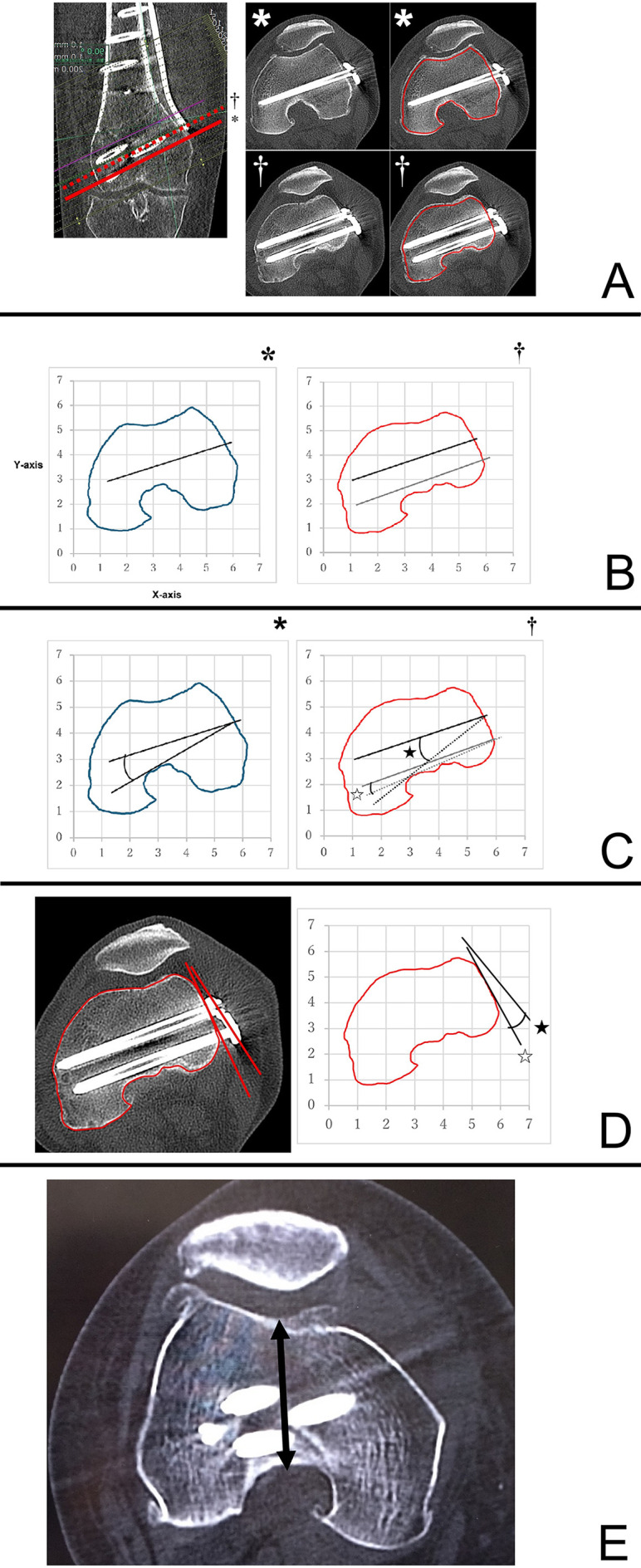
A. A curve passing through the center of the cortical bone in the cross-sectional view of the A-screw (*) and a curve passing through the center of the cortical bone in the cross-sectional view of the B–C-screw (†) were drawn. B. The curve and the lower edge of the screw were projected onto a graph. (*A-screw cross-section, †B, C-screw cross-sections). The horizontal plane was defined as the *X*-axis, and the perpendicular line was defined as the *Y*-axis. C. The maximum angle at which each screw could be adjusted without interfering with the intercondylar area is measured (*A-screw angle: AnA; † B-screw angle: AnB, denoted graphically by black star; C-Screw angle: AnC, denoted graphicallyby white star). D. On the cross-section depicting the B, C-screw, the angle between the line connecting the insertion point of the B, C-screw of the plate (denoted by black star) and the tangent line to the bony cortex (denoted by white star) was defined as AnP. E. The distance (D; denoted graphically by biconditional arrow sign ⇔) from the center of the intercondylar space to the sulcus was measured on a transverse section perpendicular to the femoral axis, depicting the transcondylar axis.

The transverse sections were constructed in a plane parallel to the three distal screws. The most distal screw was designated A, the second anterior distal row was designated B, and the second posterior distal row was designated C. As with previously described methods [13], we used the ImageJ 1.54d analysis software (Rasband, W.S., ImageJ, U.S. National Institutes of Health, Bethesda, MD) to plot curves parallel to and across the full length of each screw through the center of the femoral cortex in the cross-section of the femur for both the MCWDFO and LCWDFO groups (Figure 2A). The horizontal plane was set as the *X*-axis of the graph, and the perpendicular line to the *X*-axis was set as the *Y*-axis (Figure 2B). The maximum angle at which the lower edge of each screw makes the maximum contact with the intercondylar region without penetration was designated as (AnA), (AnB), and (AnC) for A-, B-, and C-screws, respectively (Figure 2C). Cross-sectional images were also created depicting the B and C screws, and the angle between the line connecting the B and C-screw insertion points on the plate and the tangent line to the medial and lateral bone cortex was defined as AnP (Figure 2D).

**Figure 3:**
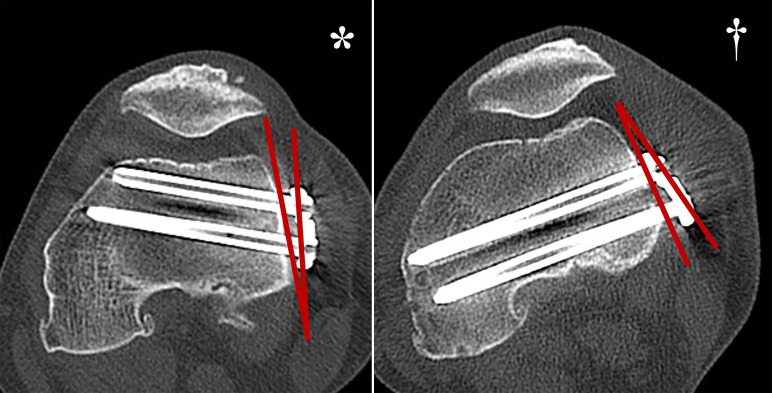
When the slope of the line connecting the B and C-screw insertion points was large relative to the tangent line to the bony cortex, it was marked as positive (*), and when it was small, it was marked as negative (†).

A large slope of the line connecting the B and C-screw insertion points relative to the cortical tangent line was defined as positive (Figure 3; marked by an asterisk), and a small inclination relative to the cortical tangent line was defined as negative (Figure 3; marked by a dagger). Cross-sectional images were also created depicting the epicondylar axis perpendicular to the femoral axis, and the distance from the sulcus to the center of the intercondylar space (D: mm) was measured (Figure 2E).

An unpaired t-test was used for the analysis, with the Bonferroni correction used to correct for multiplicity. The statistical significance level was set at 5% on both sides, and analysis was performed using SPSS 24.0 (IBM Japan, Ltd., Tokyo, Japan).

## Results

The mean values of demographic data in the MCWDFO group were as follows: age, 55.3 ± 10.1 years; height, 160.5 ± 17.2 cm; weight, 60.0 ± 10.8 kg; correction angle, 6.5 ± 2.1°. In the LCWDFO group, the mean values were as follows: age, 68.9 ± 9.9 years; height, 157.8 ± 15.3 cm; weight, 65.6 ± 14.9 kg; correction angle, 10.7 ± 2.1° (Table 1).

**Table 1:** Demographic data of the series.

Characteristics	Total
MCWDFO group	
Number of participants (*N*)	30 knees of 26 patients
Sex (male/female)	Male: 6; Female: 24
Side (left/right)	Left: 14; Right: 16
Mean age (range)	55.3 ± 10.1 (43–80)
Height (cm)	160.5 ± 17.2 (138–179)
Weight (kg)	60.0 ± 10.8 (36–88)
Correction angle	6.5 ± 2.1 (3–11)
LCWDFO group	
Number of participants (*N*)	17 knees of 17 patients
Sex (male/female)	Male: 6; Female: 11
Side (left/right)	Left: 9; Right: 8
Mean age (range)	68.9 ± 9.9 (40–79)
Height (cm)	157.8 ± 15.3 (142–177)
Weight (kg)	65.6 ± 14.9 (51–105)
Correction angle	10.7 ± 2.1 (8–13)

In the MCWDFO group, the mean values for items of evaluation were as follows: preoperative mLDFA, 83.8 ± 2.1°; postoperative mLDFA, 90.1 ± 1.4°; AnA, 10.9 ± 5.4°; AnB, 27.0 ± 4.2°; AnC, 9.2 ± 3.4°; AnP, −2.6 ± 6.9°; D, 35.7 ± 3.7 mm. In terms of gender differences, only D and postoperative mLDFA were significantly larger in males. In the LCWDFO group, the mean values for items of evaluation were as follows: preoperative mLDFA, 89.6 ± 1.9°; postoperative mLDFA, 85.2 ± 1.7°; AnA, 18.2 ± 6.9°; AnB, 30.4 ± 7.1°; AnC, 16.1 ± 7.2°; AnP, -0.2 ± 6.1°; D, 34.5 ± 3.0 mm. Only D was significantly larger in males (Table 2).

**Table 2:** Radiographic data.

Variables	Total	Male	Female	*P*-value (Male vs. Female)
MCWDFO group				
Preoperative mLDFA	83.8 ± 2.1	84.0 ± 1.1	83.8 ± 2.3	0.831
Postoperative mLDFA	90.1 ± 1.4	88.8 ± 1.6	90.4 ± 1.2	0.014
AnA	10.9 ± 5.4	11.2 ± 6.0	10.3 ± 3.8	0.273
AnB	27.0 ± 4.2	27.8 ± 4.6	26.6 ± 4.1	0.301
AnC	9.2 ± 3.4	10.3±3.8	8.7 ± 3.2	0.092
AnP	−2.6 ± 6.9	−2.5 ± 7.1	−2.7 ± 6.7	0.225
D	35.7 ± 3.7	40.0 ± 4.0	34.7 ± 2.7	0.001
LCWDFO group				
Preoperative mLDFA	89.6 ± 1.9	89.8 ± 1.6	83.8 ± 2.3	0.831
Postoperative mLDFA	85.2 ± 1.7	85.0 ± 1.6	90.4 ± 1.2	0.014
AnA	18.2 ± 6.9	19.1 ± 6.6	17.4 ± 7.1	0.387
AnB	30.4 ± 7.1	31.6 ± 4.6	29.4 ± 4.1	0.301
AnC	16.1 ± 7.2	17.5 ± 3.8	14.9 ± 3.2	0.092
AnP	−0.2 ± 6.1	−0.1 ± 7.3	−0.3 ± 6.3	0.225
D	34.5 ± 3.0	36.4 ± 3.3	33.5 ± 2.2	0.001

Next, we measured the correlation between AnA, B, and C, in addition to the correlation between all other factors. In both the MCWDFO and LCWDFO groups, AnA, B, and C correlated individually with each other, and a positive correlation was also observed with AnP. In the LCWDFO group, a positive correlation was observed with postoperative mLDFA (Table 3).

**Table 3:** Correlation between A, B, C, and each variable.

	AnA		AnB		AnC	
	*r*	*P*-value	*r*	*P*-value	*r*	*P*-value
MCWDFO group						
AnA	−	−	0.738	0.000	0.810	0.000
AnB	0.738	0.000	−	−	0.853	0.000
AnC	0.810	0.000	0.853	0.000	−	−
AnP	−0.443	0.030	−0.421	0.021	−0.412	0.019
D	0.115	0.546	0.065	0.733	0.295	0.113
Age	−0.225	0.232	−0.124	0.515	−0.329	0.076
Preoperative mLDFA	−0.189	0.316	−0.201	0.287	0.051	0.774
Postoperative mLDFA	−0.294	0.115	−0.341	0.065	−0.270	0.150
LCWDFO group						
AnA	−	−	0.946	0.000	0.933	0.000
AnB	0.946	0.000	−	−	0.966	0.000
AnC	0.933	0.000	0.966	0.000	−	−
AnP	−0.458	0.019	−0.442	0.022	−0.469	0.028
D	−0.221	0.393	−0.191	0.462	−0.302	0.239
Age	−0.029	0.912	−0.082	0.754	−0.329	0.076
Preoperative mLDFA	0.107	0.683	0.219	0.398	0.085	0.746
Postoperative mLDFA	0.643	0.05	0.560	0.019	0.579	0.015

## Discussion

When considering the direction of screw insertion on the transverse plane, the condylar morphology on the transverse plane must also be taken into consideration. The mean anteroposterior lengths of the medial and lateral femoral condyles in normal Asian knees, measured using 3D-CT, have been reported as follows: overall medial and lateral, 61.43 mm and 62.11 mm, repectively [14]; male medial and lateral, 62.6 mm and 64.8 mm, respectively; female medial and lateral, 56.4 mm and 57.8 mm, respectively [15]; male medial and lateral 67.4 mm and 68.9 mm, respectively; female medial and lateral 60.7 mm and 62.2 mm [16]. MRI has also been reported to show medial and lateral lengths of 63.36 mm, 64.38 mm [17] Overall, previous reports suggest that the medial-lateral anteroposterior length is roughly the same [14–17].

**Figure 4:**
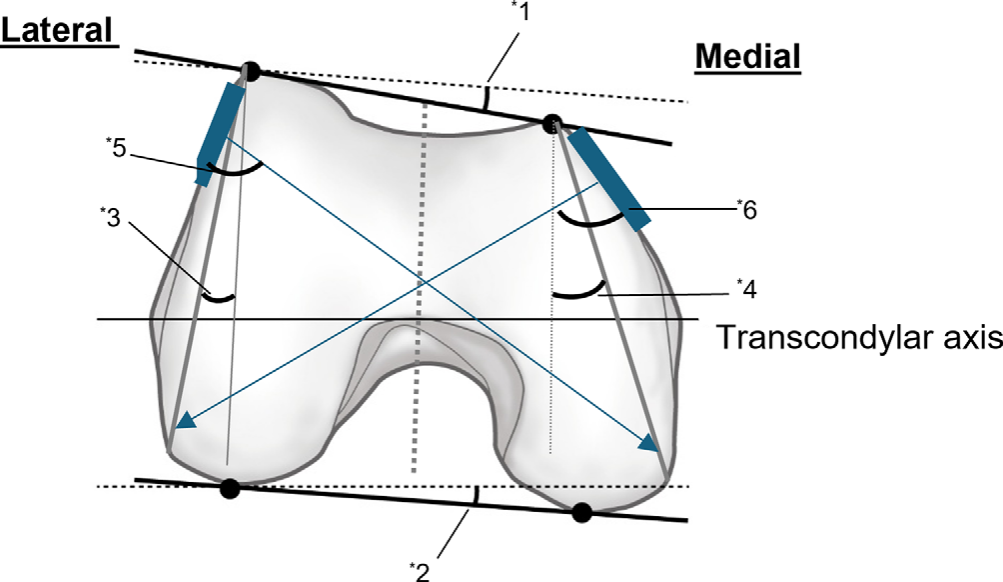
Using the posterior condylar axis as a reference, the anterior of the medial side is located posterior to the lateral side. The specific anterior edge line of the posterior slope angle with respect to the posterior condylar axis has been reported to be approximately 5.2–10 degrees (^*^1). The actual surgical position is anterior to the femoral condyle; therefore, when viewed in a cross-section, it is parallel to the SEA, which is the knee flexion/extension axis. Considering that the SEA is externally rotated approximately 3–5 degrees from the posterior condylar axis (^*^2), the anteromedial side is thought to be located further back. When compared to the intercondylar axis, several reports have described a steep slope of approximately 10 degrees on the lateral side (^*^3) and approximately 25 degrees on the medial side (^*^4) with a trapezoidal shape and a narrow anterior width and wide posterior width in the axial plane. Because the lateral plate is positioned further anteriorly than the medial plate with a smaller mediolateral slope, the screw is inserted more anterior-to-posterior on the lateral plate than the medial plate (^*^5, 6). Therefore, a longer screw may be inserted if the lateral plate is directed further posteriorly.

When assessing the anterior and posterior lengths of the condyle using horizontal MRI images – taking the total length of the condyle and the epicondylar axis as boundaries – no significant differences in overall length are noted between the medial and lateral aspects. However, if the epicondylar axis is defined as the boundary, the medial anterior segment is shorter, while the posterior segment is longer [18]. Additionally, referencing the posterior condylar axis reveals that the medial side is positioned further posteriorly relative to the lateral side, with the angle of posterior inclination of the anterior aspect reported to range from approximately 5.2–10° [14, 17] Given that the posterior condylar axis exhibits an internal rotation of about 3–5° relative to the epicondylar axis [18, 19], it is inferred that the anterior border is inclined more medially and posteriorly compared to the epicondylar axis (Figure 4).

Furthermore, when the sagittal plane (intercondylar axis) serves as the reference, existing reports suggest a pronounced medial slope of approximately 10° laterally and around 25° medially, with the horizontal section manifesting a trapezoidal shape characterized by a narrower anterior width and wider posterior width (Figure 4) [20–22].

Based on the observations above, both the anterior and posterior edges of the lateral condyle, where the distal screw is inserted, are located more anteriorly than the medial condyle. Additionally, the slope on the medial side is less pronounced on the lateral side. As a result, it is anticipated that the plate will be positioned in a more anterior and forward-facing orientation relative to the transcondylar axis on the lateral side compared to the medial side, with the morphologically flat surface facilitating forward-facing placement. Accordingly, directing the screw insertion trajectory further posterior relative to the plate surface may enable the use of longer screws (Figure 4).

**Figure 5:**
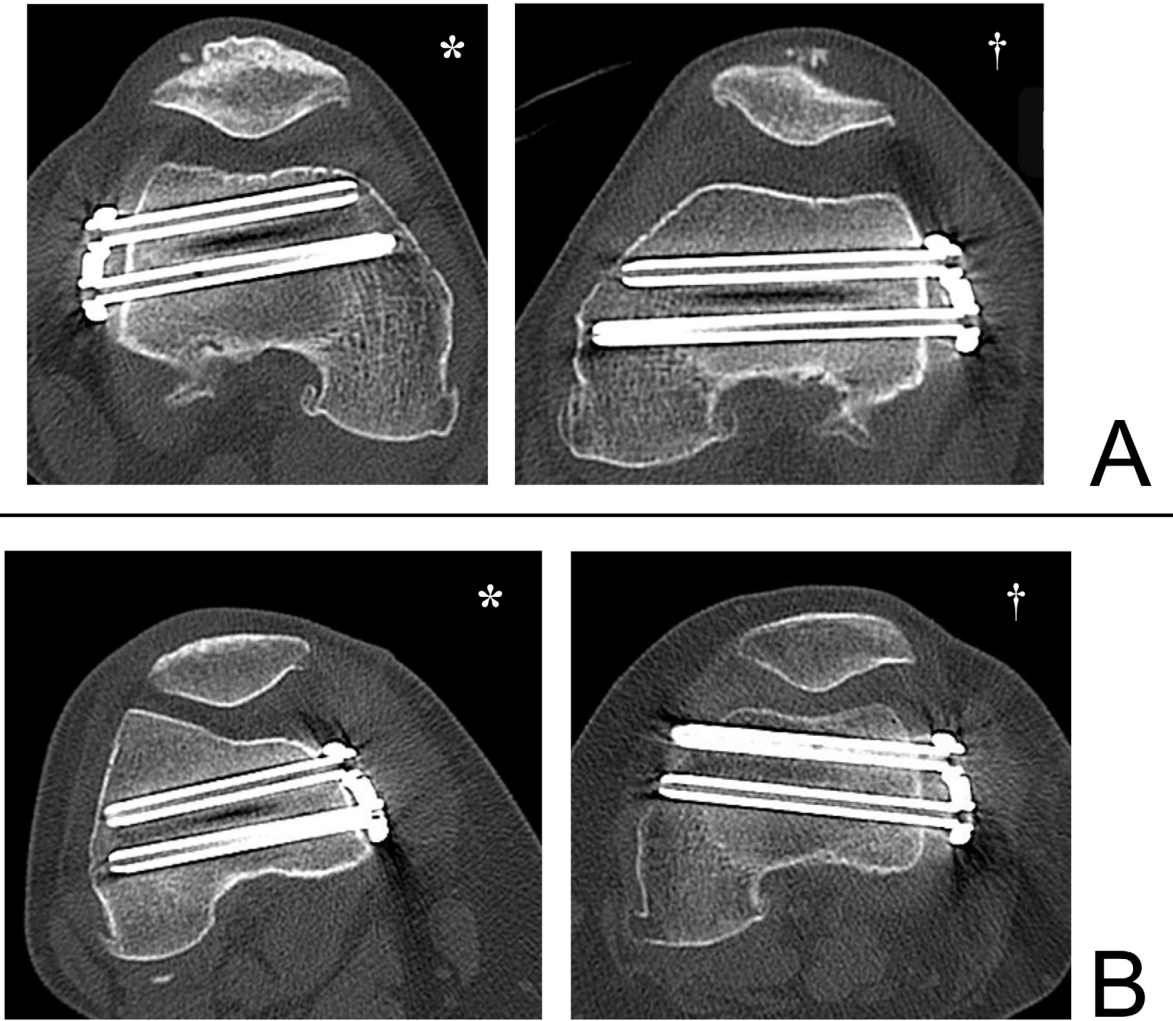
A. Differences in screw insertion direction due to differences in plate placement angle in the right image, the plate positioning angle is larger than the positioning surface; therefore, the screw is inserted anteriorly (*). On the left side, the plate positioning angle is smaller than the positioning surface; therefore, the screw is inserted posteriorly, allowing a longer screw to be inserted (†). B. With plates inserted from the medial side, the cortical surface is inclined, making it easier for the screw to be directed posteriorly to the optimal position (*); however, with plates inserted from the lateral side, the cortical surface is flat; therefore, it is important to be careful not to tilt the plate anteriorly. Implants with screws directed posteriorly may allow the screw to be inserted further (†).

This study identified a posterior margin of swing of 16–18° for the A- and C-screws and approximately 30° for the B-screw on the lateral side. Conversely, on the medial side, the placement surface exhibits a posterior inclination relative to the transcondylar axis, and the plate is typically positioned nearly parallel to this surface. In this study, the plate swing angle on the medial side generally permitted optimal positioning; however, there remains an average margin of approximately 9–11° for the A- and C-screws, and approximately 27° for the B-screw. The plate installation angle also appears to play a role in these outcomes. When comparing bilateral cases, the right side – where the installation angle exceeds the plate surface – is typically inserted more anteriorly, yielding a shorter achievable screw length (Figure 5A).

Moreover, the lateral surface’s relatively flatter profile compared to the medial side may facilitate a more anterior orientation of the plate. While inevitable due to osseous morphology, anterior plate placement results in correspondingly anterior screw insertion. Thus, increasing the posterior direction of screw insertion on the lateral plate could permit the use of longer screws than currently possible (Figure 5B).

There are several limitations to this study. First, the small sample size and single ethnicity of this study may affect the generalizability of our findings. Second, only one internal fixation plate design was evaluated. Third, the study was limited to two-dimensional analysis, which may not fully reflect the complexity of three-dimensional anatomy. Fourth, we were unable to evaluate postoperative bony union, analyze differences in screw insertion direction, or assess clinical outcomes. Finally, even when screws were inserted in the optimal trajectory, some instances involved screws of insufficient length that did not reach the contralateral cortex. For future research, we plan to compare clinical outcomes between cases with screws inserted optimally and to maximum length and those in which these criteria were not met.

## Conclusions

Both the medial and lateral plates for DFO internal fixation can be positioned approximately 10 degrees posteriorly on the medial side and approximately 15 degrees posteriorly on the lateral side compared to the current distal screw insertion angle, which may allow for the insertion of longer screws.

For DFO internal fixation plates, we believe that inserting the distal screw from anterior to posterior enables greater insertion length and improved fixation. On the medial aspect, the surface inclines posteriorly with respect to both the epicondylar and posterior condylar axes. The plate positioning was nearly parallel to the surface, resulting in near-optimal positioning; however, the orientation of the A- and C-screws may potentially be adjusted posteriorly by approximately 9–11°, and the B-screw by approximately 27°. Conversely, the lateral surface is flatter with reduced inclination; therefore, the plate does not lie parallel to the surface, causing the anterior side to float and leading to a more forward-facing insertion of the screws. Consequently, it may be feasible to adjust the orientation of the A- and C-screws further posteriorly compared to the medial side by approximately 16–18°, and the B screw by approximately 30°.

## Data Availability

The data that supports the findings of this study are included in this article. Further inquiries can be directed to the corresponding author.
